# Optimizing patient check-in process for telehealth visits: a data-driven perspective

**DOI:** 10.3389/fdgth.2025.1554762

**Published:** 2025-05-12

**Authors:** Kunal Khashu

**Affiliations:** HCA Healthcare, Nashville, TN, United States

**Keywords:** artificial intelligence, check in check out, digital health, patient portal, advanced analytics, decision support system, telehealth

## Introduction

The expansion of telehealth services has revolutionized healthcare delivery, offering patients greater convenience and providers enhanced operational flexibility (1). Despite this progress, the patient check-in process remains a critical point of inefficiency, often undermining the overall telehealth experience. Challenges such as unclear instructions, incomplete patient data, and inefficient communication persist, creating barriers to seamless virtual care. Addressing these issues requires a systematic approach leveraging decision support systems (DSS) and patient-centered digital innovations to enhance process efficiency and patient satisfaction.

The patient check-in process is not just an administrative step but a crucial determinant of patient engagement and provider efficiency. Optimizing this process can significantly impact the success of telehealth by improving patient outcomes, reducing no-shows, and fostering a more cohesive care delivery model (2). In this article, we explore the pressing challenges in telehealth check-ins, propose solutions grounded in data-driven strategies, and discuss their implications for the future of virtual care.

## Key challenges and proposed solutions

**Figure 1:**
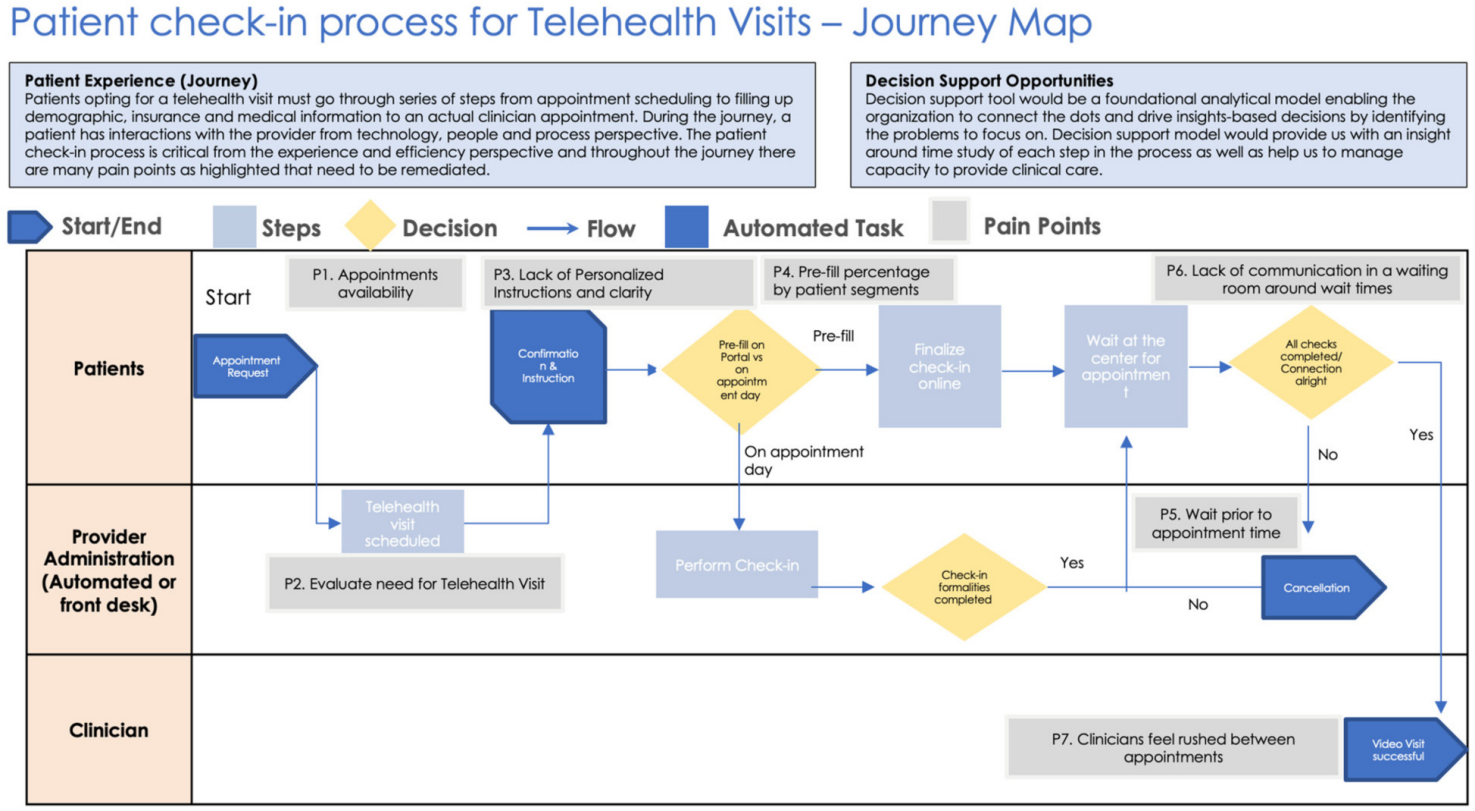
Patient check-in process overview highlighting pain points across the process.

One of the most significant challenges in telehealth check-in processes is fragmented pre-visit preparation. Many patients fail to complete necessary pre-check-in formalities, leading to delays and disruptions during virtual visits (Figure 1, Table 1). This issue is often exacerbated by unclear or overly complex instructions, which can result in incomplete documentation and miscommunication between patients and healthcare providers. Additionally, gaps in communication create uncertainty, as patients receive little to no updates regarding wait times and process statuses, leaving them frustrated and disengaged (Figure 1, Table 1). The inefficiency in data collection further complicates the process, as patients are frequently required to enter redundant information due to poor integration between electronic health records (EHR) and telehealth platforms. Furthermore, technical barriers disproportionately affect older adults and individuals with limited digital literacy, hindering their ability to navigate telehealth systems effectively (3).

**Table 1:** Key to the patient check-in process flow as per Figure 1 describing pain points.

#	Pain point	Description	Interaction point
P1	Appointment availability	Due to the talent shortages, demand sometimes outweighs supply and hence there are many instances where a patient is not able to secure an appointment on a desired date due to lack of availability of appointments	Online Portal, Phone, Chat
P2	Evaluate need for Telehealth Visit	A framework to determine eligibility of a patient for a telehealth visit prior to scheduling an appointment would allow freeing up capacity for the patients who are eligible	Portal, Chat, Survey questions, Checklist
P3	Lack of Personalized Instructions and clarity	Once appointment is booked, an instruction email is sent over to patients prompting them to complete the pre-appointment steps. This is a critical step that and our target is to have >90% compliance. A decision support tool would help us to measure the percentage compliance by patient segments based on various attributes.	Email
P4	Pre-fill percentage by patient segments	Insights into compliance of patients who prefill the information would help us to tailor the instructions and follow up framework to drive greater compliance	Data
P5	Wait prior to appointment time	For the patients who have prefilled their information, arrived ahead of time, there has to be an integrated scheduling process to map the patients with the available clinicians as quickly as possible	Waiting room, Front desk
P6	Lack of communication in a waiting room around wait times	Patients waiting have no means to be made aware that how long is the wait time.	Front desk or Kiosk
P7	Clinicians feel rushed between appointments	Back to back scheduling for clinicians create little lag between appointments and clinicians always feel rushed to their next appointment	Scheduling system

**Figure 2:**
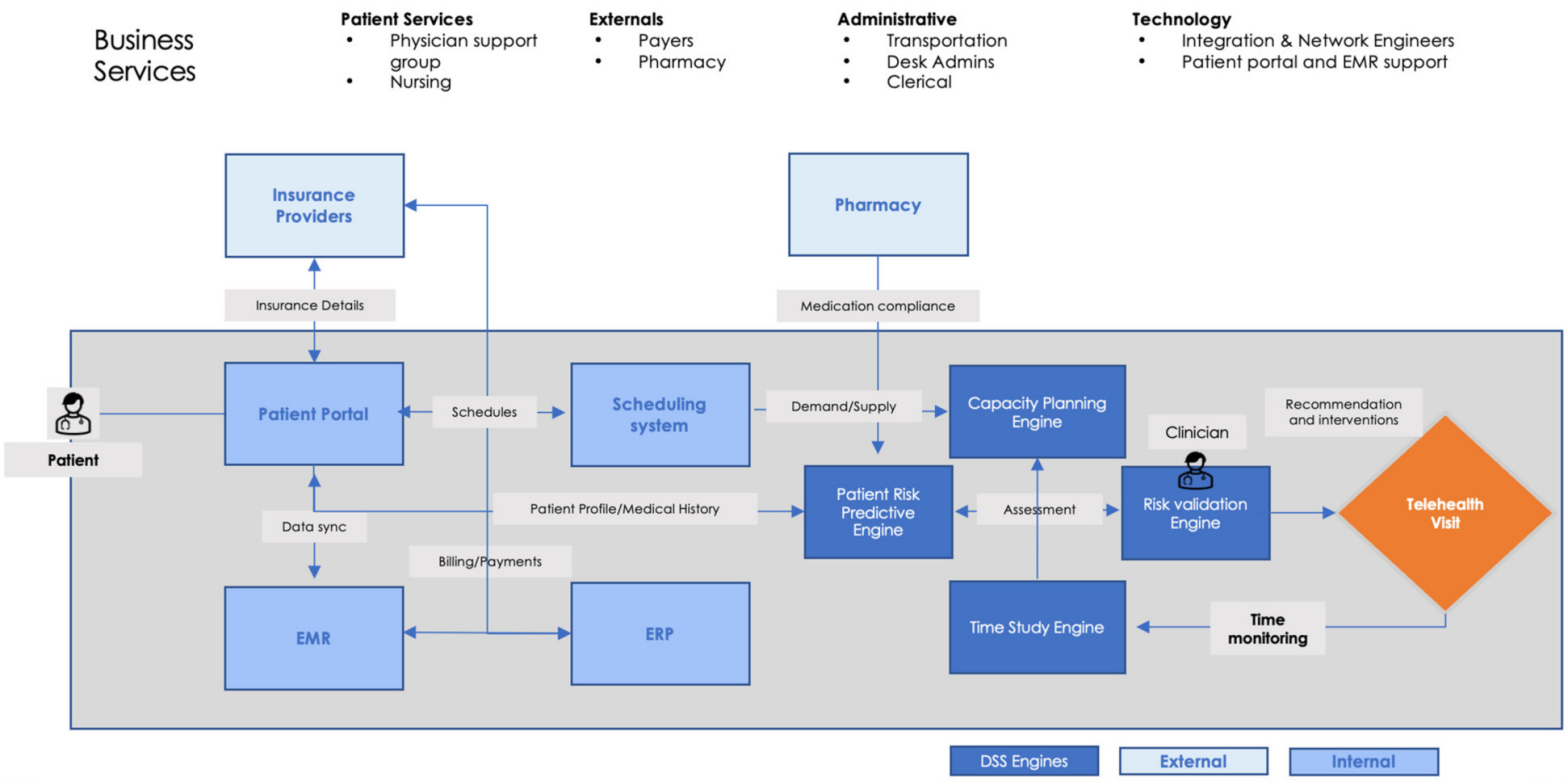
Proposed business context diagram highlighting role of decision support systems.

To address these challenges, healthcare organizations must implement data-driven solutions that enhance efficiency and patient experience (Figure 2). Automated pre-visit reminders and instructional guides powered by artificial intelligence can ensure that patients complete check-in requirements before their scheduled appointments, reducing delays and minimizing administrative errors (Table 2) (4). The integration of patient portals with telehealth systems can streamline the check-in workflow by automatically retrieving and updating patient information, eliminating repetitive data entry. Real-time status updates and notifications can enhance communication between patients and providers, alleviating concerns about wait times and connectivity issues. Artificial intelligence-powered virtual assistants can further support patients by guiding them through the check-in process, answering questions, and troubleshooting common technical difficulties. Additionally, personalized digital workflows that adapt to a patient's medical history, demographic information, and technical proficiency can improve adherence to check-in protocols and optimize overall telehealth accessibility.

**Table 2:** Methods and techniques to enable patient experience and operational efficiencies.

Methods	Member/patients	Innovation capability	Resources	Techniques
When a patient logs on to the Portal for scheduling an appointment for a Telehealth Visit, there needs to be a validation against existing EMR records, health plans as well as available public records to pre-populate relevant information such as Insurance details, demographic, medical history etc.	Patient Information database	Attribute	Provider Integrated systems	Capture relevant data by integration patient portal with the internal (EMR) and external sources. Integration needs to be seamless and bi-directional to maintain data integrity in case of any updates by the patient on the portal. Ex: A patient can add a phone number or change an address on a portal. Seamless integration must ensure the data is updated in a system of record
Data captured in the first step on a member portal could be assessed to create a patient profile that could be made available to the patients through all the back-end integrations. Patients could schedule an appointment online through the portal.	Patient Profile	Describe	Appointment Scheduler	Leverage the patient profile captured on a patient portal to prioritize appointments for telehealth visits by mapping the patient with available and appropriate clinicians.
Leverage predictive analytics and machine learning to create a risk profile by synching data such as medication outcomes, support system at home, prior admissions etc.	Patient Risk Predictive Engine	Predict	Provider Risk assessment & validation Engine	Leverage Machine learning and predictive analytics to map risk profiles to suggest targeted and tailored programs and interventions to a patient as a preventive well being initiative during the telehealth visit.
Capture time study data of patients from check-in to check-out and create a forecasting model leveraging patient and risk profile to ascertain visit time for patients and variability by risk profile.	Time Study Engine	Prescribe	Capacity Planning Engine	Based on the number of patients, patient and risk profile and leverage forecasting techniques to perform capacity planning for the clinicians and staffs that would be required to meet he demand. Ensure the capacity plan is updated on regular intervals based on variability of demand.
Anytime, anywhere communication support such as patient portal, chat (Virtual and Live) so that patient could reach out through a preferred communication channel and vice versa	Communication channels	Orchestrate	Feedback management	Leveraging communication channels to provide necessary support, communication and gather feedback to continuously improve the overall process

## Decision support systems: a foundational framework

Decision support systems (DSS) offer a robust framework to analyze and optimize the telehealth check-in process. By integrating predictive analytics, time-study engines, and capacity planning tools, DSS enables healthcare organizations to improve operational efficiency and enhance patient satisfaction (Figure 2).

A crucial advantage of DSS is process time analysis, which allows healthcare providers to track the time taken for each step in the check-in process. By analyzing this data, organizations can identify bottlenecks and inefficiencies that hinder patient experience. Understanding the specific areas where delays occur enables targeted interventions that streamline workflows and improve service delivery.

Resource allocation is another key function of DSS in telehealth. By analyzing demand patterns and provider availability, DSS helps optimize staffing and resource distribution. Ensuring adequate staffing levels based on patient volume reduces unnecessary wait times and enhances overall patient satisfaction. Effective resource planning, backed by data-driven insights, allows healthcare organizations to allocate personnel efficiently and respond dynamically to fluctuations in demand.

Personalized recommendations generated by DSS enhance the patient experience by delivering customized instructions and reminders. By leveraging historical patient data, demographic characteristics, and medical history, DSS can tailor communication to the specific needs of each patient. This level of personalization improves adherence to check-in protocols, reduces errors, and increases engagement, ultimately leading to better patient outcomes.

Proactive issue resolution is another significant benefit of DSS, as it enables real-time identification of potential technical or procedural issues. By continuously monitoring system performance and patient interactions, DSS can detect issues such as connectivity problems, incomplete documentation, or scheduling conflicts. Early detection allows healthcare providers to intervene swiftly and prevent disruptions that could negatively impact the patient experience. This proactive approach ensures a smoother and more reliable telehealth check-in process.

For healthcare providers, implementing a DSS not only enhances operational efficiency but also supports a patient-centric approach that prioritizes convenience and clarity. The integration of DSS within telehealth systems allows organizations to make data-driven decisions that optimize processes, reduce inefficiencies, and improve overall service quality.

## Leveraging technology for seamless integration

Technology serves as a crucial enabler in optimizing the telehealth check-in process by addressing inefficiencies, reducing administrative burdens, and enhancing the overall patient experience. A well-integrated digital infrastructure facilitates smoother interactions between patients and healthcare providers, ensuring that check-in procedures are efficient and user-friendly.

One of the primary technological advancements in telehealth check-in is the implementation of patient portals (Tables 2, 3). These platforms empower patients to complete pre-check-in tasks such as submitting medical history, insurance details, and consent forms from the convenience of their own homes, reducing wait times and administrative bottlenecks upon arrival. Features such as auto-fill capabilities, intuitive interfaces, and step-by-step guidance improve user engagement and completion rates. By streamlining these processes, healthcare providers can allocate resources more effectively, focusing on patient care rather than administrative paperwork.

**Table 3:** Methods and techniques elaboration table elaborating various proposed methods and techniques.

Method/technique	Description and
Patient Risk Predictive Engine	• **What:** The Patient Risk predictive engine would leverage machine learning algorithms to analyze data and make predictions about potential medical conditions of a patient. • **How:** The engine would leverage the patient data stored in the EMR or collected through a patient portal as well as external systems such as medication, pharmacy, demographic etc. to create a risk profile of a patient. The engine will be a cloud-based solution build on a platform that would be procured/bought from the vendor. • **Why:** This would be foundational to enable more preventive and proactive approach to care. The risk profile created would enable clinicians to design a targeted programs and interventions
Provider Risk assessment & Validation Engine	• **What:** Provider Risk assessment engine would map risk profiles to suggest targeted and tailored programs and interventions to a patient and would provide a baseline to clinicians to validate and finalize • **How:** The engine would leverage the risk profiles from the Patient Risk predictive engine as an input and leverage machine learning to map potential risks with a targeted diagnostics or intervention programs. The engine will be built and integrated with the system of record standard solutions such as EMR • **Why:** The mapping of risk profile with the targeted programs would enable clinicians to validate the baseline and suggest recommendations to patients faster than a traditional approach. This would enable clinicians as well as patients to deploy preventive and holistic approach to care
Time Study Engine	• **What:** Time study of patients of all types and risk profiles from patient check-in to patient check-out to be leveraged in a forecasting model to predict the timings for future visits and improve the capacity plan. • **How:** The engine would leverage the risk profiles, historical visitation time as an input and forecasting models to provide predictions around total time from check-in to check-out by risk profiles and patient profiles, The forecasting model would be created in-house • **Why:** Time study engine would be critical to enhance capacity planning by predicting demand and visitation hours and mapping clinicians appropriately.
Capacity Planning Engine	• **What:** The engine will leverage patient data, targeted programs and clinician schedule to forecast demand and predict capacity required to fulfil care delivery. • **How:** The engine will be procured from a vendor and configured to meet the demands of a provider. The engine will integrate with wide variety of internal systems such as EMR, scheduling system, member portal to be able to size the demand up and predict capacity required. • **Why:** The capacity planning engine is critical to ensure providers can meet the demand and deliver care to the patients.
Communication Channels and Feedback management	• **What:** Patient and clinician engagement layer that would allow two-way communication through online portal and chat between the patients and clinicians • **How:** The engine would leverage online portal with virtual and live chat capabilities. The engine will be procured from the vendor and configured based on the needs of the providers. • **Why:** The communication channels would enable clinicians to provide support to patients outside the premises and help patients to access care anywhere anytime. The channels will have feedback mechanisms that would allow clinicians to gather crucial feedback data to continuously improve the process.

Automated communication systems (Tables 2, 3) further enhance the telehealth check-in experience by ensuring patients receive timely and personalized notifications. These systems, which leverage email, SMS, or app-based messaging, provide reminders about upcoming appointments, instructions on necessary pre-visit preparations, and real-time updates regarding scheduling changes. Such automation reduces patient uncertainty and decreases the likelihood of missed appointments, thereby improving overall operational efficiency for healthcare providers.

Real-time support solutions, such as chatbots and AI-driven virtual assistants, contribute to a seamless telehealth check-in experience by addressing common patient queries without requiring human intervention. Integrated into patient portals, these tools can assist with troubleshooting technical issues, answering frequently asked questions, and guiding patients through the check-in process. By providing immediate assistance, these solutions alleviate pressure on administrative staff and improve patient satisfaction.

Another critical component of technology-driven telehealth integration is interoperability. Seamless connectivity between electronic medical records (EMRs), scheduling systems, and billing platforms ensures that data flows efficiently across multiple systems, eliminating redundant manual data entry and reducing the risk of errors. Interoperability enables healthcare providers to access up-to-date patient information in real time, ensuring continuity of care and improving clinical decision-making (Table 4) (5).

**Table 4:** Business context diagram elaboration table describing proposed DSS frameworks.

DSS engine	Systems/integrations	Description
Patient Risk Predictive Engine	Pharmacy Integration	In order to create a risk profile for a patient, we need to obtain the medication compliance history from the pharmacy.
	Medical History	Integration needs to be built between Patient Risk predictive engine and the existing EMR systems to obtain medical and diagnostic history of a patient. This integration is critical to create a risk profile of a patient
	Patient Profile	Engine would be integrated with the Patient portal to obtain the patient profile consisting of demographic, insurance and medical history data to create a risk profile
Risk Validation Engine	Patient Risk Predictive Engine Interface	Risk Validation engine is an analytical front end of the Patient Risk predictive engine that would provide an access to clinicians to access the risk profile of a patient before the telehealth appointment. This interface would also map the risk profile with relevant interventions and programs, allowing clinicians to focus on holistic care of the patient during telehealth visit.
Capacity Planning Engine	Scheduling System Integration	This is a demand planning engine that would map the supply (Existing schedules) with a demand (Patient Portal) to create the list of appointments that would be offered to patients through a portal. The engine would optimize itself based on the Time study results through an integration with a Time study engine.
Time Study Engine	Data collection from Telehealth Visits	Data would be collected from each step during a telehealth visit to identify the optimized time model required for telehealth visit.
	Capacity Planning Engine Interface	Optimized time would be fed into the capacity planning engine to determine the throughput

The integration of these technological solutions not only enhances the efficiency of telehealth check-in but also fosters a more patient-centered approach to healthcare delivery. By leveraging digital tools, healthcare providers can reduce administrative burdens, improve data accuracy, and create a more accessible and convenient experience for patients. As telehealth continues to expand, adopting and refining these technologies will be essential to ensuring a seamless and effective check-in process.

## Proposed business context diagram

### Recommended solution implications for patient experience

Optimizing the telehealth check-in process has significant implications for patient experience, influencing satisfaction, engagement, and overall perception of care quality. A seamless and efficient check-in establishes a positive first impression, fostering trust and confidence in telehealth services. Patients who encounter minimal friction during the check-in process are more likely to feel valued and engaged, leading to higher satisfaction levels and long-term loyalty to their healthcare providers.

One of the most direct benefits of an improved telehealth check-in process is the reduction of appointment cancellations and no-shows. Confusing or inefficient check-in procedures often discourage patients from completing necessary pre-visit requirements, which can lead to missed appointments and scheduling inefficiencies. By implementing user-friendly digital interfaces, automated reminders, and real-time status updates, healthcare providers can minimize barriers to engagement and ensure patients arrive prepared for their virtual consultations. Streamlined pre-visit processes also enhance accessibility, particularly for individuals with limited digital literacy or technical proficiency, making telehealth a more viable and inclusive option for diverse patient populations.

Beyond logistical benefits, an optimized check-in system enhances the perceived value of telehealth services. Patients are more likely to recognize the convenience and efficiency of virtual care when they experience a frictionless check-in process that mirrors or exceeds the ease of traditional in-person visits. A well-integrated digital workflow ensures that patients have access to the necessary information, such as pre-visit instructions, estimated wait times, and technical support, fostering a sense of preparedness and empowerment.

Empowering patients through an optimized check-in process also encourages greater participation in their own healthcare. By providing clear guidance, intuitive self-service options, and AI-driven assistance, healthcare providers can support patients in actively managing their health information and medical appointments. Access to real-time updates, seamless integration with patient portals, and the ability to complete administrative tasks in advance enable patients to engage more proactively in their care journey. When patients feel more in control and informed about their healthcare interactions, they are more likely to adhere to treatment plans, communicate effectively with providers, and maintain long-term engagement with telehealth services.

Ultimately, a well-designed telehealth check-in process not only benefits healthcare providers by improving operational efficiency but also significantly enhances the overall patient experience. By addressing common pain points and leveraging technology-driven solutions, healthcare organizations can foster stronger patient-provider relationships, promote higher levels of trust and satisfaction, and reinforce the value of virtual care as an integral component of modern healthcare delivery.

## Operational benefits for providers

From an operational standpoint, optimizing the telehealth check-in process yields significant advantages for healthcare providers by improving efficiency, reducing costs, and enhancing data quality. The automation of repetitive administrative tasks minimizes the workload on healthcare staff, enabling them to focus on higher-value activities such as direct patient care and complex case management. By leveraging artificial intelligence and digital tools, healthcare organizations can streamline appointment scheduling, patient verification, and pre-visit documentation, resulting in a more efficient workflow. This reduction in administrative burden not only enhances staff productivity but also contributes to a more seamless patient experience.

In addition to improving efficiency, a well-integrated telehealth check-in system generates substantial cost savings for healthcare organizations. Inefficiencies in manual processes, such as redundant data entry and appointment scheduling conflicts, often lead to increased operational expenses. By automating these processes and integrating digital check-in solutions with electronic medical records (EMRs), providers can optimize resource allocation, reduce labor costs, and minimize the likelihood of errors that could result in financial losses. Improved patient adherence to pre-visit procedures also decreases the frequency of missed appointments and last-minute cancellations, further contributing to cost containment and revenue optimization.

Another key operational benefit of a streamlined check-in process is the enhancement of data quality. Accurate and complete patient records are essential for effective clinical decision-making, and integrating data collection with EMRs ensures that healthcare providers have access to reliable and up-to-date patient information. Automated check-in systems reduce the risk of data discrepancies by minimizing manual entry errors and ensuring consistency across different healthcare platforms. Additionally, improved data accuracy facilitates better coordination among providers, supporting more informed decision-making and ultimately leading to improved patient outcomes.

By addressing inefficiencies in the check-in workflow, healthcare organizations can achieve a more sustainable telehealth model that benefits both providers and patients. Streamlining administrative processes, reducing operational costs, and ensuring high-quality data integration create a more efficient and effective telehealth ecosystem. As digital transformation continues to reshape healthcare delivery, optimizing the telehealth check-in process remains a critical factor in achieving long-term success in virtual care.

## Challenges and considerations

While optimizing the telehealth check-in process offers numerous benefits, the implementation of these solutions presents several challenges that healthcare organizations must address. Adoption barriers, privacy concerns, and scalability issues can hinder the effectiveness of digital transformation efforts in telehealth. Successfully overcoming these challenges requires a strategic approach that includes comprehensive training, robust cybersecurity measures, and scalable infrastructure.

One of the primary challenges in implementing a streamlined telehealth check-in process is resistance to adoption (3). Patients and healthcare staff may be hesitant to embrace new digital systems due to unfamiliarity, perceived complexity, or concerns about usability. Many patients, particularly older adults and those with limited digital literacy, may struggle with navigating new interfaces or completing self-service check-ins (6). Similarly, healthcare staff may resist adopting automated workflows if they feel the transition will disrupt established processes or require extensive retraining. To mitigate these barriers, healthcare organizations must invest in user-friendly system designs, provide ongoing training, and offer real-time support to ensure both patients and staff can confidently use the new technology.

Privacy concerns represent another critical consideration in telehealth check-in optimization. The increasing reliance on digital platforms for patient intake and medical record management necessitates stringent cybersecurity protocols to protect sensitive health information. Compliance with regulations such as the Health Insurance Portability and Accountability Act (HIPAA) is essential to maintaining patient trust and avoiding legal repercussions. Healthcare organizations must implement end-to-end encryption, multi-factor authentication, and continuous monitoring systems to safeguard patient data. Additionally, transparent communication about data security measures can help reassure patients about the safety of their personal information, increasing their willingness to engage with digital check-in tools.

Scalability is another major factor to consider when implementing digital check-in solutions. As telehealth services continue to expand, digital platforms must be capable of accommodating increasing patient volumes and diverse healthcare use cases without compromising performance. A poorly scalable system can lead to slow processing times, technical glitches, and patient frustration, ultimately undermining the benefits of automation. To address this challenge, healthcare providers should invest in cloud-based solutions, modular software designs, and interoperability with existing electronic medical records (EMRs) to ensure seamless integration and efficient scalability. By designing flexible systems that can adapt to evolving patient needs, organizations can future-proof their telehealth infrastructure and sustain long-term operational success.

Addressing these challenges requires a proactive and strategic approach that balances innovation with practicality. Healthcare organizations must prioritize usability, security, and scalability to ensure that digital check-in solutions enhance, rather than hinder, the patient experience. By implementing comprehensive training programs, enforcing robust data protection measures, and designing scalable infrastructure, providers can maximize the benefits of telehealth while mitigating potential risks.

## Future directions

As telehealth continues to evolve, the future of check-in processes will likely be shaped by advancements in artificial intelligence (AI), the integration of wearable health technologies, and a greater emphasis on equitable healthcare access. These innovations have the potential to significantly enhance patient engagement, optimize clinical workflows, and ensure inclusivity in telehealth services. By adopting these forward-thinking solutions, healthcare providers can better meet the changing needs of patients in an increasingly digital healthcare landscape.

AI-driven personalization is expected to play a central role in the next generation of telehealth check-in systems. Advanced algorithms powered by machine learning can analyze patient data to deliver personalized recommendations, automate pre-visit questionnaires, and provide tailored health interventions. AI-powered virtual assistants can further enhance the patient experience by guiding users through the check-in process, answering questions, and flagging potential issues before the appointment. These personalized interactions can improve patient adherence to pre-visit protocols, reduce administrative burdens, and foster a more engaging telehealth experience.

Another key area of future development is the integration of wearable health devices into telehealth workflows. Wearable technology, such as smartwatches, continuous glucose monitors, and remote blood pressure cuffs, provides real-time health data that can be incorporated into check-in procedures. This integration allows clinicians to review up-to-date physiological metrics before the appointment, enabling more informed decision-making and proactive intervention when necessary. For instance, remote monitoring of heart rate variability or oxygen saturation levels can help physicians assess a patient's health status before the consultation, leading to more personalized and effective care.

An essential consideration for the future of telehealth check-in processes is the adoption of equity-focused design principles. Despite technological advancements, disparities in healthcare access persist, particularly among underserved populations. To bridge this gap, telehealth platforms must prioritize accessibility by incorporating multilingual interfaces, intuitive navigation, and compatibility with a range of devices, including lower-cost smartphones and tablets. Additionally, targeted outreach and digital literacy training can help ensure that vulnerable populations, such as elderly patients and individuals in rural areas, can effectively use telehealth services. By designing telehealth solutions with inclusivity in mind, healthcare providers can promote equitable access to care and mitigate disparities in digital health adoption.

By embracing these future innovations, healthcare organizations can stay ahead of technological advancements and continue to enhance patient experiences in the digital era. The convergence of AI-driven personalization, wearable device integration, and inclusive design principles will shape the next phase of telehealth, enabling a more efficient, accessible, and patient-centered approach to virtual care. As these technologies evolve, continuous research and adaptation will be necessary to ensure their successful implementation and widespread adoption.

## Conclusion

The optimization of telehealth check-in processes presents a transformative opportunity to enhance patient experience, improve operational efficiency, and drive future innovation in digital healthcare. By addressing key challenges such as fragmented pre-visit preparation, inefficiencies in data collection, and technological barriers, healthcare providers can create a seamless check-in experience that fosters patient engagement and trust. Implementing AI-driven automation, real-time communication, and decision support systems ensures a streamlined workflow, reducing administrative burdens while enhancing clinical decision-making.

From an operational standpoint, refining the telehealth check-in process translates into tangible benefits for providers. Automated workflows and improved resource allocation result in cost savings, reduced staff workload, and enhanced data quality, ultimately supporting better patient outcomes. The integration of advanced digital tools not only minimizes inefficiencies but also empowers patients by providing clear instructions and personalized recommendations, strengthening their role in their own care journey.

Looking ahead, the future of telehealth check-in processes will be shaped by AI-driven personalization, wearable health technology, and an equity-focused approach to digital healthcare. By leveraging predictive analytics, real-time health data, and accessible telehealth platforms, healthcare providers can ensure that virtual care remains efficient, inclusive, and responsive to patient needs. However, successful implementation requires overcoming adoption barriers, safeguarding patient data, and ensuring scalable solutions that can accommodate increasing demand.

Ultimately, optimizing telehealth check-in processes is not just an operational improvement but a strategic imperative in the evolving healthcare landscape. As digital health technologies continue to advance, healthcare organizations must remain agile in their approach, continuously refining their systems to enhance efficiency, accessibility, and patient satisfaction. By embracing innovation and prioritizing patient-centric design, providers can drive lasting improvements in telehealth delivery, ensuring high-quality, equitable, and seamless virtual care experiences for all patients.
